# Supplementary material for: Effectiveness of Breastfeeding Support Packages in Low- and Middle-Income Countries for Infants under Six Months: A Systematic Review
Source: Nutrients. 2021 Feb 20;13(2):681. doi: 10.3390/nu13020681 (PMC7924359; doi:10.3390/nu13020681)
Supplement: Supplementary file 1 [file nutrients-13-00681-s001.pdf]

Table S1.1 Effect of breastfeeding interventions (n=37) on breastfeeding practices

No.	Author, population, study quality	Intervention	Outcome	Direction of effect
**COUNSELLING (n=11)**				
1	Ahmadi et al., 2016<br>Mothers with premature (34-37wk) infants<br>(+,++)	**Breastfeeding consultation**<br>IG: Consultation sessions based on BASNEF model (5 sessions of 30 min, 5 consecutive days)<br>CG: Conventional training by staff<br>Delivery: Hospital-based	**Breastfeeding practices (IG vs CG)**<br>Comparison of continuation of EBF (%), baseline,1,2,3,4m	↑
2	Albernaz et al., 2003<br>Infants<br>(+,+)	**Lactation counselling**<br>-based on 40-h WHO lactation support training course<br>IG: Support by trained nurse-hospital and home visits (5,15,30,45,60,90 and 120d)<br>CG: Standard care<br>Delivery: Hospital-based	**Breastfeeding practices (IG vs CG)**<br>EBF compared to fully weaned at 4m, % [40 vs 31]	↔
3	Tahir & Al-Sadat, 2013<br>Mothers<br>(-,+)	**Telephone lactation counselling**<br>IG: Counselling twice monthly by certified lactation counsellors + conventional care<br>CG: Conventional care<br>Delivery: Hospital-based	**Breastfeeding practices (IG vs CG)**<br>EBF at 6m, RR(CI) [1.04(0.58-1.87)]	↔
4	Aidam et al., 2005<br>Pregnant women attending prenatal clinics<br>(+,+)	**Lactation counselling**<br>-based on WHO/UNICEF counselling training manual, LINKAGES- “facts for feeding” brochure, Helping Mothers to Breastfeed-book<br>IG1: Pre, peri and postnatally<br>IG2: Peri and postnatally<br>CG: Non-BF health education support<br>Delivery: Hospital-based	**Breastfeeding practices (IG1, IG2 vs CG)**†<br>Comparison of continuation of EBF (%) from baseline-1-2-3-4-5-6m (over the previous 24h)	↑
5	Oliveira et al., 2014<br>Adolescent mothers with newborns and their mothers<br>(+,+)	**Counselling sessions**<br>-based on WHO guiding principles<br>IG1: AG not living with mother, with int<br>CG1: AG not living with mother, without int<br>IG2: AG living with mother, with int<br>CG2: AG living with mother, without int<br>Delivery: Hospital-based	**Breastfeeding practices**<br><u>Kaplan-Meier curve, EBF (birth-1-2-3-4-5-6m)</u><br>EBF (IG1 vs CG1)<br>EBF (IG2 vs CG2)	 <br>↑<br>↑
6, 7	Kimani-Murage et al., 2016; Kimani-Murage et al., 2017	**Home-based nutrition counselling**<br>IG: CHWs were trained to offer counselling on MIYCN<br>CG: Standard care	**Breastfeeding practices (IG vs CG)**<br><u>EBF for 0-6m, %(CI)</u><br>IG [55.2(50.4-59.9)] vs CG [54.6(50.0-59.1]	 <br>↔

No.	Author, population, study quality	Intervention	Outcome	Direction of effect
	Mother-child pairs<br>(++,++)	Delivery: Community-based		
8, 9	Engebretsen et al., 2014<br>Tylleskar et al., 2011<br>Mother-infant pairs<br>(++,++)	**EBF counselling**<br>-by peer counsellors from local communities trained in 1wk WHO courses-Breastfeeding Counselling: A training course, and HIV and Infant Feeding Counselling: A training course<br>IG: Peer counselling (1 antenatal and 4 postnatal)<br>CG: Usual care<br>Delivery: Community-based	**Breastfeeding practices (IG vs CG), Burkina Faso**<br>EBF, PR(CI)<br>24wks, 24-h recall [3.33(1.74, 6.38)]<br>**Breastfeeding practices (IG vs CG), Uganda**<br>EBF, PR(CI)<br>24wks, 24-h recall [3.83(2.97, 4.95)]<br>**Breastfeeding practices (IG vs CG), South Africa**<br>EBF, PR(CI)<br>24wks, 24-h recall [5.70(1.33, 24.56)]	↑<br>↑<br>↑
10	Morrow et al., 1999<br>Pregnant women<br>(-,-)	**Home based peer counselling**<br>-by trained counselor (La Leche League Mexico)<br>IG1: 6 visits, IG2: 3 visits<br>CG: No intervention<br>Delivery: Community-based	**Breastfeeding practices (IG1 and IG2 vs CG)**[†]<br>Duration of any BF ≥6m, % [87 vs 76]	↔
11	Leite et al., 2005<br>Mothers-infants<br>(+,-)	**Home based peer counselling**<br>IG: Home visits 5,15,30,60,90 and 120d<br>CG: Standard care<br>Delivery: Community-based	**Breastfeeding practices (IG vs CG)**<br>Feeding practices at 4m, %<br>EBF [24.7 vs 19.4]	↑
**EDUCATION (n=8)**				
12	Froozani et al., 1999<br>Mother-infant pairs<br>(-,+)	**BF education**<br>IG: Education, face-to-face, after delivery and during follow-up<br>CG: Usual care<br>Delivery: Hospital-based	**Breastfeeding practices (IG vs CG)**<br>Feeding pattern 4m after delivery (%)<br>EBF [54 vs 6.5]	↑
13	Neyzi et al., 1991<br>Mother-infant pairs<br>(-,-)	**Educational intervention**<br>IG: 2 sessions after delivery<br>CG: Usual care<br>Delivery: Hospital-based	**Breastfeeding practices (IG vs CG)**<br>EBF at 4m, % [67.7 vs 5.0]	↑
14	Khresheh et al., 2011<br>Primiparous women<br>(+,+)	**Educational programme supporting BF**<br>IG: One-to-one postnatal education sessions and follow-up phone calls at 2 and 4m pp<br>CG: Routine care<br>Delivery: Hospital-based	**Breastfeeding practices (IG vs CG)**<br>BF only at 6m, % [39 vs 27]	↔
15	Susin & Giugliani, 2008<br>Mother-father-infant triads<br>(+,+)	**Educational session by a trained paediatrician**<br>-18 minutes video, discussion, handouts<br>IG1: Intervention to mother+father	**Breastfeeding practices**<br>Discontinuation of EBF at 6m, HR(CI)<br>IG1 vs CG [0.65(0.45-0.96)]	↑

No.	Author, population, study quality	Intervention	Outcome	Direction of effect
		IG2: Intervention to mother only<br>CG: No intervention<br>Delivery: Hospital-based	IG2 vs CG [0.93(0.66-1.31)]	↔
16	Ozluses & Celebioglu, 2014<br>Couples with their infants<br>(-,-)	**Educating fathers**<br>IG1: Mothers-20min/d<br>IG2: Mothers+fathers-20min/d<br>CG: No EBF education<br>Delivery: Hospital-based	**Breastfeeding practices (IG1 vs IG2 vs CG)**<br>EBF at 6m,% [33.3 vs 56.4 vs 12.8]	↑
17	Su & Ouyand, 2016<br>Pregnant women<br>(-,-)	**Breastfeeding education**<br>IG: Education to mother+father<br>CG: Education to mother only<br>Delivery: Hospital-based	**Breastfeeding practices (IG vs CG)**<br>EBF at 6m, % [40.0 vs 17.6]	↑
18	Aksu et al., 2011<br>Pregnant women<br>(+,+)	**Postnatal BF education**<br>-Standard BF education to both groups in antenatal period<br>-Based on 18h WHO/UNICEF BF counselling/lactation management course<br>IG: BF education at home on day 3 postpartum (reinforcement)<br>CG: No education/support<br>Delivery: Community-based	**Breastfeeding practices (IG vs CG)**<br>EBF at 6m, % [43 vs 23]<br>Duration of EBF, m [4.7±1.2 vs 3.9±1.2]	↑<br>↑
19	Jakobsen et al., 2008<br>Mothers and infants<br>(+,+)	**Health education**<br>-based on WHO recommendations<br>IG: Education provided individually and orally in local language<br>CG: Standard care<br>Delivery: Community-based	**Breastfeeding practices (IG vs CG)**<br><u>Time of introduction of water, HR(CI)</u><br>121-180d [0.18(0.06-0.49)]	↑
**TRAINING (n=7)**				
20	Agrasada et al., 2005<br>Mother-infant (term LBW) pairs<br>(+,-)	**Training programme for counsellors**<br>IG1: BF counselling by trained counsellors<br>IG2: Child feeding counselling by trained counsellors<br>CG: Any counselling<br>Delivery: Hospital-based	**Breastfeeding practices (IG1, IG2 vs CG)**[†]<br>Comparison of EBF from 2wk-6m	↑
21	Khayyati & Mansouri, 2009<br>Pregnant women<br>(-,-)	**Training movies**<br>-important BF points and ways for doing<br>IG: Training movies and common method of face-to-face training<br>CG: Face-to-face training	**Breastfeeding practices (IG vs CG)**<br>EBF at 6m, % [49.1 vs 51.2]	↔

No.	Author, population, study quality	Intervention	Outcome	Direction of effect
		Delivery: Hospital-based		
22	Bhandari et al., 2003<br>Infants<br>(++,++)	**Promotion of EBF by training existing primary healthcare health and nutrition workers**<br>-based on IMNCI training manual on BF counselling<br>IG: Promotion by CHWs trained in BF (3-day course)<br>CG: Usual care<br>Delivery: Community-based	**Breastfeeding practices (IG vs CG)**<br>EBF at 6m, OR(CI) [17.6(6.6-47.2)]	↑
23	Balaluka et al., 2012<br>Infants<br>(+,+)	**Trained CVs**<br>-part of community-based nutrition project where CVs were trained in BF<br>IG: Promoting EBF via door-to-door visits and community meetings<br>CG: Usual care only<br>Delivery: Community-based	**Breastfeeding practices (IG vs CG)**<br>EBF at 6m, % (CI) [57.7(50.9-64.5) vs 2.7(1.1-6.6)]	↑
24	Mukantwali et al., 2006<br>Mother-infant pairs<br>(-,-)	**Special training of CHWs**<br>IG: Visited by specially trained CHW<br>CG: Visited by normally trained CHW<br>Delivery: Community-based	**Breastfeeding practices (IG vs CG)**<br>EBF at 6m (7d recall), % [67 vs 37]	↔
25	Mukhopadhyay et al., 2017<br>Mother-infant pairs<br>(-,-)	**Modular training on infant feeding to CHWs in local vernacular**<br>IG: Trained CHWs<br>CG: Standard care<br>Delivery: Community-based	**Breastfeeding practices (IG vs CG)**<br>EBF at 6m, % [79 vs 64]	↑
26	Shamim et al., 2017<br>Mothers of infants <6m<br>(+,+)	**Special training**<br>-5d training course (WHO/UNICEF breastfeeding counselling training guidelines)<br>IG1: Trained TBAs/CVs<br>IG2: Trained + supervised TBAs/CVs<br>CG: TBAs/CVs without special training<br>Delivery: Community-based	**Breastfeeding practices**‡<br><u>EIBF within 1h (pre-post), %</u><br>IG1 [38→60]<br>IG2 [35→68]<br>CG [29→35]<br><u>EBF 24h prior to interview (pre-post), %</u><br>IG1 [63→76]<br>IG2 [62→83]<br>CG [61→67]	 <br>↑<br>↑<br>↑<br><br>↔<br>↔<br>↔
**PROMOTION (n=4)**				
27	Cangol & Sahin, 2017<br>Pregnant- applied to pregnancy preparation course<br>(-,+)	**BF motivation programme based on Pender's Health Promotion Model**<br>IG: 4 times-antenatal period, 1st postnatal day, 4th-6th postnatal wk and 4th postnatal month<br>CG: Standard care	**Breastfeeding practices (IG vs CG)**<br>Intention to BF, % [94.1 vs 75.8]	↑

No.	Author, population, study quality	Intervention	Outcome	Direction of effect
		Delivery: Hospital-based		
28	Gu et al., 2016<br>Primiparous women companied by husband/ mother<br>(-,+)	**Theory of Planned Behaviour (TPB)-based intervention programme**<br>IG: Individual instruction, group education and telephone counselling<br>CG: Routine nursing care<br>Delivery: Hospital-based	**Breastfeeding practices (IG vs CG)**<br>BF at 6m, PD(CI) [31.9(22.0, 40.7)]	↑
29	Moudi et al., 2016<br>Primiparous women referred to health centre<br>(+,-)	**BF promotion**<br>IG1: Peer support group (4 times)<br>IG2: Health care provider's education (4 training sessions)<br>CG: Routine care<br>Delivery: Hospital-based	**Breastfeeding practices (IG1 vs IG2 vs CG)**<br><u>Duration of EBF, mean(sd)</u><br>EBF at 8wk [21.7±24.44 vs 10.10±16.23 vs 8.5±14.93]	↑
30	Akram et al., 1997<br>Pregnant women<br>(-,-)	**Promotion of EBF**<br>IG: Health messages<br>CG: No health messages<br>Delivery: Community-based	**Breastfeeding practices (IG vs CG)**<br>Prelacteal feeds, % [66 vs 31]	↑
**PEER SUPPORT (n=4)**				
31, 32, 33	Bich & Cuong, 2017; Bich et al., 2016; Bich et al., 2014<br>Fathers and their pregnant wives from 7-30 wk gestation<br>(+,+)	**Fathers as supporters**<br>IG: BF education material, counselling services at community health centres, invitation to social events and household visits<br>CG: No intervention to fathers<br>Delivery: Community-based	**Breastfeeding practices (IG vs CG)**<br><u>At 6m, %</u><br>EBF (since birth) [6.7 vs 0.9]	↑
34	Dearden et al., 2002<br>Mothers of infants <6m<br>(+,+)	**Mother-to-mother support programme of La Leche League Guatemala**<br>IG: BF counselling by trained counsellor<br>CG: Usual care<br>Delivery: Community-based	**Breastfeeding practices**<br><u>Changes in IG vs CG from baseline (%)</u><br>EIBF, within 1h [+1.0 vs +3.0]<br>EBF in past 24-h [+2.1 vs -3.9]	 <br>↓<br>↑
**OTHER INTERVENTION (n=3)**				
35	Yotebieng et al., 2015<br>Mother-infant pairs<br>(++,++)	**10 steps of successful BF programme**<br>IG1: BFHI steps 1-9<br>IG2: BFHI steps 1-10<br>CG: Standard care<br>Delivery: Hospital-based	**Breastfeeding practices**<br><u>EIBF, PR(CI)</u><br>IG1 vs CG [1·01(0·79-1·30)]<br>IG2 vs CG [0·98(0·73-1·32)]<br><u>EBF at 24 wks, PR(CI)</u><br>24-h recall IG1 vs CG [3·50(2·76-4·43)]<br>24-h recall IG2 vs CG [1·31(0·91-1·89)]	 <br>↔<br>↔<br><br>↑<br>↔

No.	Author, population, study quality	Intervention	Outcome	Direction of effect
36	Menon et al., 2016<br>Infants<br>(++,++)	**BF practices at scale**<br>IG: Intensified IPC, MM, CM, and PA<br>CG: Standard nutrition counselling and less intensive MM, CM, and PA<br>Delivery: Community-based	**Breastfeeding practices (IG vs CG), Bangladesh**<br><u>DDE between baseline and endline, %(CI)</u><br>EIBF, within 1h of birth [16.70(2.78, 30.57)]<br>EBF, infants <6m [36.20(21.01, 51.46)]<br>**Breastfeeding practices (IG vs CG), Vietnam**<br><u>DDE between baseline and endline, %(CI)</u><br>EIBF, within 1h of birth [10.00(-1.25, 21.40)]<br>EBF, infants <6m [27.90(17.74, 38.07)]	 <br>↑<br>↑<br><br><br>↔<br>↑
37	Ochola et al., 2013<br>Pregnant women (34-36 wk) attending antenatal clinic<br>(+,+)	**Counselling on EBF**<br>IG1: Home based intensive counselling group (HBIC)-7 sessions at home by trained peers (1 prenatally and 6 postnatally)<br>IG2: Facility based semi-intensive counselling group (FBSIC)-1 session prenatally	**Breastfeeding practices (IG1, IG2 vs CG)**<br>Comparison of EBF, RR(CI)<br><u>EBF at 6m</u><br>HBIC vs CG [4.20(1.66-10.64)]<br>FBSIC vs CG [1.64(0.56- 4.81)]	 <br><br>↑<br>↔

Table S1.2 Effect of breastfeeding interventions (n=12) on caregivers' knowledge/skills/practices

No.	Author, population, study quality	Intervention	Outcome	Direction of effect
**COUNSELLING (n=1)**				
1	Ahmadi et al., 2016<br>Mothers with premature (34-37wk) infants<br>(+,++)	**Breastfeeding consultation**<br>IG: Consultation sessions based on BASNEF model (5 sessions of 30 min, 5 consecutive days)<br>CG: Conventional training by staff<br>Delivery: Hospital-based	**Caregivers knowledge/skills/practices (IG vs CG)**<br>Comparison of mean scores of structures in BASNEF model (1-knowledge, 2-attitude, 3-subjective norms, 4-enabling factors, 5-outcome evaluation) from baseline-1-2-3-4m<br>Comparison of mean lactation performance score, baseline,1,2,3,4m	 ↑<br><br><br>↑
**EDUCATION (n=4)**				
2	Khresheh et al., 2011<br>Primiparous women<br>(+,+)	**Educational programme supporting BF**<br>IG: One-to-one postnatal education sessions and follow-up phone calls at 2 and 4m pp<br>CG: Routine care<br>Delivery: Hospital-based	**Caregivers knowledge/skills/practices (IG vs CG)**<br>Knowledge of BF before and after at 6m, % (7 components)	 ↕
3	Su & Ouyand, 2016<br>Pregnant women<br>(-,-)	**Breastfeeding education**<br>IG: Education to mother+father<br>CG: Education to mother only<br>Delivery: Hospital-based	**Caregivers knowledge/skills/practices[‡]**<br><u>Comparison of BF knowledge (before-after)</u><br>Mean score IG [61.73→87.51], CG [72.23→87.04]<br><u>Comparison of BF attitude (before-after)</u><br>Mean score IG [59.14→66.50], CG [61.81→66.98]	 <br>↑, ↑<br><br>↑, ↑
4	Ahmed, 2008<br>Mothers and preterm infants (born <37wk)	**Educational Programme**<br>-based on PRECEDE (Predisposing, Reinforcing, Enabling Constructs in Educational Diagnosis and Evaluation) model	**Caregivers knowledge/skills/ practices[‡]**<br><u>Comparison of mean knowledge scores (pre- and post-test)</u><br>IG [28.87±12.19→68.73±8.80]	 <br>↑

No.	Author, population, study quality	Intervention	Outcome	Direction of effect
	(-,-)	IG: 5 session<br>CG: Routine care<br>Delivery: Hospital-based	CG [24.47±4.79→40.00±6.66]	↑
5	Aksu et al., 2011<br>Pregnant women<br>(+,+)	**Postnatal BF education**<br>-Standard BF education to both groups in antenatal period<br>-Based on 18h WHO/UNICEF BF counselling/lactation management course<br>IG: BF education at home on day 3 postpartum (reinforcement)<br>CG: No education/support<br>Delivery: Community-based	**Caregivers knowledge/skills/practices (IG vs CG)**<br>Mean BF knowledge scores at 6wk [5.8±1.7 vs 4.8±1.3]	↑
**PROMOTION (n=4)**				
**Hospital-based**				
6	Cangol & Sahin, 2017<br>Pregnant- applied to pregnancy preparation course<br>(-,+)	**BF motivation programme based on Pender's Health Promotion Model**<br>IG: 4 times-antenatal period, 1st postnatal day, 4th-6th postnatal wk and 4th postnatal month<br>CG: Standard care<br>Delivery: Hospital-based	**Caregivers knowledge/ skills/practices (IG vs CG)**<br>Self-efficacy, mean(sd) [8.12±2.19 vs 7.36±1.93]	↔
7	Saljughi et al., 2016<br>Pregnant women<br>(-,-)	**Role playing for promotion of BF**<br>IG: Training on promoting BF self-efficacy at 36th wk via role playing<br>CG: Routine care<br>Delivery: Hospital-based	**Caregivers knowledge/ skills/practices (IG vs CG)**<br><u>Comparison, mean(sd) scores</u><br>Self-efficacy [13.2±6.9 vs 3.3±4.01]	↑
8	Akram et al., 1997<br>Pregnant women<br>(-,-)	**Promotion of EBF**<br>IG: Health messages<br>CG: No health messages<br>Delivery: Community-based	**Caregivers knowledge/ skills/practices (IG vs CG)**<br><u>Knowledge, %</u><br>Advantage of giving colostrum [74 vs 55]<br>EIBF [66 vs 31]	 <br>↑<br>↑
9	Reinsma et al., 2016<br>Pregnant women and their partners<br>(+,-)	**Audio programme (Bobbi Be Best) and discussion guide to promote EBF**<br>IG: Entertainment education-unique 15 minutes episode about EBF followed by a discussion led by local health worker (once a week, for a total 4wks)<br>CG: Entertainment education-unique 15 minutes episode about injection safety followed by a discussion led by health worker (once a week, for a total 4wks)<br>Delivery: Community-based	**Caregivers knowledge/ skills/ practices (IG vs CG)[‡]**<br><u>Pre- and post-test comparison, median scores</u><br>Knowledge of EBF [10→10] vs [10→10]<br>Misconception about EBF [14→10] vs [18→14]<br>Barriers to EBF [6→6] vs [12→10]<br>Benefits of EBF [26→30] vs [27→26]<br>Self-efficacy [20→20] vs [20→20]<br>Intention to EBF [5→5] vs [5→5]	 <br>↔<br>↑, ↑<br>↔<br>↑, ↔<br>↔<br>↔
**PEER SUPPORT (n=3)**				
10, 11, 12	Bich & Cuong, 2017; Bich et al., 2016; Bich et al., 2014	**Fathers as supporters**<br>IG: BF education material, counselling services at community health centres, invitation to social events and household visits	**Caregivers knowledge/skills/practices (IG vs CG)**<br>Parental knowledge score, mean difference [6.3]<br>Parental attitude score, mean difference<br>EIBF [1.7]	 ↑<br><br>↑

No.	Author, population, study quality	Intervention	Outcome	Direction of effect
	Fathers and their pregnant wives from 7-30 wk gestation<br>(+,+)	CG: No intervention to fathers<br>Delivery: Community-based	EBF [1.3]<br>Supporting mothers to BF [-0.01]<br>Total attitude score [2.9]	↑<br>↔<br>↑

Table S1.3 Effect of breastfeeding interventions (n=2) on healthcare staffs' knowledge/skills/practices

No.	Author, population, study quality	Intervention	Outcome	Direction of effect
**TRAINING (n=2)**				
1	Ma et al., 2018<br>Healthcare professional-doctor, nurse, midwife<br>(+,+)	**BF DVD training (15 minutes)**<br>-developed by researchers in England, mandarin voice-over, covers 4 sessions-optimal positioning and attachment, satisfying feed, hand expression and baby led feed<br>IG: BF essential support skills DVD<br>CG: Vaginal delivery DVD<br>Delivery: Hospital-based	**Healthcare staff knowledge/skills/practices**<br>Comparison of differences in mean(sd) knowledge scores (pre-post-test)<br>P&A (range 0-6), knowledge<br>IG [2.73±1.41→4.04±1.16], CG [2.86±1.37→2.83±1.19]<br>HE (range 0-6), knowledge<br>IG [2.66±1.09→3.70±1.00], CG [2.81±1.13→2.73±1.19]<br>Comparison of differences in mean(sd) confidence scores (pre-post-test)<br>P&A (range 0-60)<br>IG [49.93±8.48→53.90±6.93], CG [50.68±8.62→50.82±8.89]<br>HE (range 0-20)<br>IG [16.57±3.29→17.91±2.51], CG [17.59±2.85→17.49±2.77]	↑, ↔<br>↑, ↔<br>↑, ↔<br>↑, ↔
2	Shamim et al., 2017<br>Mothers of infants <6m<br>(+,+)	**Special training**<br>-5d training course (WHO/UNICEF breastfeeding counselling training guidelines)<br>IG1: Trained TBAs/CVs<br>IG2: Trained + supervised TBAs/CVs<br>CG: TBAs/CVs without special training<br>Delivery: Community-based	**Healthcare staff knowledge/ skills/practices**<br>Comparison of proportion of TBAs who knew items before and after training, %<br>EIBF [72 vs 92]<br>Prelacteal feeding [88 vs 85]<br>EBF [86 vs 94]	↑<br>↔<br>↔

Table S1.4 Effect of breastfeeding interventions (n=9) on morbidity

No.	Author, population, study quality	Intervention	Outcome	Direction of effect
**COUNSELLING (n=3)**				
1, 2	Engebretsen et al., 2014<br>Tylleskar et al., 2011<br>Mother-infant pairs<br>(++,++)	**EBF counselling**<br>-by peer counsellors from local communities trained in 1wk WHO courses-Breastfeeding Counselling: A training course, and HIV and Infant Feeding Counselling: A training course<br>IG: Peer counselling (1 antenatal and 4 postnatal)<br>CG: Usual care<br>Delivery: Community-based	**Morbidity (IG vs CG), Burkina Faso**<br>Diarrheal morbidity (2wks recall), PR(CI)<br>24wks [0.83(0.45, 1.54)]<br>**Morbidity (IG vs CG), Uganda**<br>Diarrheal morbidity (2wks recall), PR(CI)<br>24wks [0.82(0.58, 1.15)]<br>**Morbidity (IG vs CG), South Africa**<br>Diarrheal morbidity (2wks recall), PR(CI)<br>24wks [1.31(0.89,1.93)]	 <br>↔<br><br><br>↔<br><br><br>↔
3	Morrow et al., 1999<br>Pregnant women<br>(-,-)	**Home based peer counselling**<br>-by trained counselor (La Leche League Mexico)<br>IG1: 6 visits, IG2: 3 visits<br>CG: No intervention<br>Delivery: Community-based	**Morbidity**<br>Diarrhea in infants 0-3m, % [12 vs 26]	 ↑
**EDUCATION (n=3)**				
4	Froozani et al., 1999<br>Mother-infant pairs<br>(-,+)	**BF education**<br>IG: Education, face-to-face, after delivery and during follow-up<br>CG: Usual care<br>Delivery: Hospital-based	**Morbidity (IG vs CG)**<br>Number of days of illness, 4m after delivery, mean(sd)<br>Diarrheal illness [1.2(2.7) vs 4.0(7.1)]<br>Respiratory illness [3.2(5.0) vs 3.7(8.8)]	 <br>↔<br>↔
5	Khresheh et al., 2011<br>Primiparous women<br>(+,+)	**Educational programme supporting BF**<br>IG: One-to-one postnatal education sessions and follow-up phone calls at 2 and 4m pp<br>CG: Routine care<br>Delivery: Hospital-based	**Morbidity (IG vs CG)**<br>Mild illness at 6m, % [58 vs 69]<br>Hospitalisation at 6m, % [11 vs 18]	 ↔<br>↔
6	Jakobsen et al., 2008<br>Mothers and infants<br>(+,+)	**Health education**<br>-based on WHO recommendations<br>IG: Education provided individually and orally in local language<br>CG: Standard care<br>Delivery: Community-based	**Morbidity (IG vs CG)**<br>Diarrhea morbidity (no. of episodes per 100d of observation), OR(CI)<br>7-180d [0.98(0.83-1.17)]	 <br><br>↔
**TRAINING (n=2)**				
7	Khayyati & Mansouri, 2009<br>Pregnant women<br>(-,-)	**Training movies**<br>-important BF points and ways for doing	**Morbidity (IG vs CG)**<br>Infant disease at 6m, % [0.9 vs 4.8]	 ↔

No.	Author, population, study quality	Intervention	Outcome	Direction of effect
		IG: Training movies and common method of face-to-face training<br>CG: Face-to-face training<br>Delivery: Hospital-based		
8	Bhandari et al., 2003<br>Infants<br>(++,++)	**Promotion of EBF by training existing primary healthcare health and nutrition workers**<br>-based on IMNCI training manual on BF counselling<br>IG: Promotion by CHWs trained in BF (3-day course)<br>CG: Usual care<br>Delivery: Community-based	**Morbidity (IG vs CG)**<br>At 6m, OR(CI)<br>Diarrhea in previous 24h [1.05(0.81-1.36)]<br>Diarrhea in previous 7d [0.85(0.72-0.99)]<br>Diarrhea episode in previous 3m for which treatment was sought outside home [0.68(0.50-0.92)]	 <br>↔<br>↑<br>↑
**OTHER INTERVENTIONS (n=1)**				
9	Yotebieng et al., 2015<br>Mother-infant pairs<br>(++,++)	**10 steps of successful BF programme**<br>IG1: BFHI steps 1-9<br>IG2: BFHI steps 1-10<br>CG: Standard care<br>Delivery: Hospital-based	**Morbidity**<br>Diarrhea since last visit at 24 wks, PR(CI)<br>IG1 vs CG [0·50(0·34-0·73)]<br>IG2 vs CG [1·23(1·03-1·46)]<br>Fever with cough at 24 wks, PR(CI)<br>IG1 vs CG [0·84(0·43-1·62)]<br>IG2 vs CG [1·16(0·71-1·88)]	 <br>↑<br>↓<br><br>↔<br>↔

Table S1.5 Effect of breastfeeding interventions (n=11) on anthropometry

No.	Author, population, study quality	Intervention	Outcome	Direction of effect
**COUNSELLING (n=4)**				
1	Ahmadi et al., 2016<br>Mothers with premature (34-37wk) infants<br>(+,++)	**Breastfeeding consultation**<br>IG: Consultation sessions based on BASNEF model (5 sessions of 30 min, 5 consecutive days)<br>CG: Conventional training by staff<br>Delivery: Hospital-based	**Anthropometry (IG vs CG)**<br>Comparison of mean infant weight, baseline,1,2,3,4m	 ↑
2	Albernaz et al., 2003<br>Infants<br>(+,+)	**Lactation counselling**<br>-based on 40-h WHO lactation support training course<br>IG: Support by trained nurse-hospital and home visits (5,15,30,45,60,90 and 120d)<br>CG: Standard care<br>Delivery: Hospital-based	**Anthropometry (IG vs CG)**<br>Mean weight (compared (0-14-30-45-60-90-120d)	 ↔
3, 4	Engebretsen et al., 2014<br>Tylleskar et al., 2011<br>Mother-infant pairs<br>(++,++)	**EBF counselling**<br>-by peer counsellors from local communities trained in 1wk WHO courses-Breastfeeding Counselling: A training course, and HIV and Infant Feeding Counselling: A training course	**Anthropometry at 24wks (IG vs CG), Burkina Faso**<br>Mean z-scores difference (CI)<br>WLZ [-0.20(-0.39, -0.01)]<br>LAZ [-0.02(-0.20, 0.16)]<br>WAZ [-0.15(-0.34, 0.05)]	 <br>↓<br>↔<br>↔

No.	Author, population, study quality	Intervention	Outcome	Direction of effect
		IG: Peer counselling (1 antenatal and 4 postnatal)	Wasting (WLZ, <-2sd), PR(CI)	
		CG: Usual care	Wasting [1.40(0.84, 2.32)]	↔
		Delivery: Community-based	Stunting (LAZ, <-2sd), PR(CI)	
			Stunting [1.07(0.73, 1.57)]	↔
			Underweight (WAZ, <-2sd), PR(CI)	
			Underweight [1.23(0.88, 1.71)]	↔
			**Anthropometry at 24wks (IG vs CG), Uganda**	
			Mean z-scores difference (CI)	
			WLZ [-0.23(-0.43, -0.3)]	↓
			LAZ [0.13(-0.33, 0.06)]	↔
			WAZ [-0.26 (-0.44, -0.08)]	↓
			Wasting (WLZ, <-2sd), PR(CI)	
			Wasting [2.36(1.11, 5.00)]	↑
			Stunting (LAZ, <-2sd), PR(CI)	
			Stunting [1.28(0.86, 1.90)]	↔
			Underweight (WAZ, <-2sd), PR(CI)	
			Underweight [1.52(0.81, 2.88)]	↔
			**Anthropometry at 24wks (IG vs CG), South Africa**	
			Mean z-scores difference (CI)	
			WLZ [0.23 (0.03, 0.43)]	↑
			LAZ [-0.13(-0.34, 0.08)]	↔
			WAZ [0.09(-0.13, 0.30)]	↔
			Wasting (WLZ, <-2sd), PR(CI)	
			Wasting [1.12(0.30, 4.11)]	↔
			Stunting (LAZ, <-2sd), PR(CI)	
			Stunting [1.07(0.70, 1.65)]	↔
			Underweight (WAZ, <-2sd), PR(CI)	
			Underweight [1.18(0.58, 2.38)]	↔
**EDUCATION (n=2)**				
5	Froozani et al., 1999	**BF education**	**Anthropometry (IG vs CG)**	
	Mother-infant pairs	IG: Education, face-to-face, after delivery and during follow-up	Weight and length, 4m after delivery, mean(sd)	
	(-,+)	CG: Usual care	Weight, kg [6335.4(740) vs 6054.8(752)]	↑
		Delivery: Hospital-based	Length, cm [63.2(2.3) vs 62.4(1.9)]	↑
6	Jakobsen et al., 2008	**Health education**	**Anthropometry (IG vs CG)**	
	Mothers and infants	-based on WHO recommendations	Median weight (kg)	
	(+,+)	IG: Education provided individually and orally in local language	7-60d [4.7 vs 4.8]	↔
		CG: Standard care	61-120d [6.0 vs 6.0]	↔
		Delivery: Community-based	121-150d [6.8 vs 7.0]	↓
			151-180d [7.5 vs 7.8]	↓
			Weight for age, WAZ, Median z-scores	
			7-60d [0.44 vs 0.42]	↔

No.	Author, population, study quality	Intervention	Outcome	Direction of effect
			61-120d [0.45 vs 0.59]<br>121-150d [0.19 vs 0.47]<br>151-180d [-0.16 vs 0.08]	↔<br>↓<br>↓
**TRAINING (n=4)**				
7	Khayyati & Mansouri, 2009<br>Pregnant women<br>(-,-)	**Training movies**<br>-important BF points and ways for doing<br>IG: Training movies and common method of face-to-face training<br>CG: Face-to-face training<br>Delivery: Hospital-based	**Anthropometry (IG vs CG)**<br>Weight, mean(sd) [3309±481 vs 3194±4820]	 ↔
8	Bhandari et al., 2003<br>Infants<br>(++,++)	**Promotion of EBF by training existing primary healthcare health and nutrition workers**<br>-based on IMNCI training manual on BF counselling<br>IG: Promotion by CHWs trained in BF (3-day course)<br>CG: Usual care<br>Delivery: Community-based	**Anthropometry (IG vs CG)**<br>Mean weight, length<br>HAZ, WHZ (<-2sd, %)	 ↔<br>↔
9	Balaluka et al., 2012<br>Infants<br>(+,+)	**Trained CVs**<br>-part of community-based nutrition project where CVs were trained in BF<br>IG: Promoting EBF via door-to-door visits and community meetings<br>CG: Usual care only<br>Delivery: Community-based	**Anthropometry (IG vs CG)**<br>Weight at 6m, mean(sd) [6.73±1.23 vs 6.61±0.92]	 ↔
10	Mukhopadhyay et al., 2017<br>Mother-infant pairs<br>(-,-)	**Modular training on infant feeding to CHWs in local vernacular**<br>IG: Trained CHWs<br>CG: Standard care<br>Delivery: Community-based	**Anthropometry (IG vs CG)**<br>Weight, mean(sd) [3682±727 vs 3067±719]<br>Length, mean(sd) [15.22±3.7 vs 14.64±3.0]	 ↑<br>↔
**PROMOTION (n=1)**				
11	Cangol & Sahin, 2017<br>Pregnant- applied to pregnancy preparation course<br>(-,+)	**BF motivation programme based on Pender's Health Promotion Model**<br>IG: 4 times-antenatal period, 1st postnatal day, 4th-6th postnatal wk and 4th postnatal month<br>CG: Standard care<br>Delivery: Hospital-based	**Anthropometry (IG vs CG)**<br>Birth weight(kg), mean(sd) [3.35±0.47 vs 3.19±0.40]	 ↔

Footnote:

Symbols used- ↑, positive effect; ↓, negative effect; ↔, no effect; ↕ mixed effect; †, analysis included comparison of IG1/IG2 with CG; ‡, analysis included before after comparison in intervention and control

Abbreviations- AG, adolescent girls; BASNEF, beliefs, attitudes, subjective norms and enabling factors; BHFI, baby friendly hospital initiative; CG, control group; CHW, community health worker; CI, confidence interval; CM, community mobilisation; CV, community volunteer; d, day; DDE, difference-in-difference estimate; EBF, exclusive breastfeeding; EIBF, early initiation of breastfeeding; GATHER, greet clients, ask clients about themselves, tell clients about their choices, help clients choose, explain what to do and return for follow up; gs, gestation; h, hour; HAZ, height-for-age z-score; HE, hand expression; HR, hazard ratio; IG, intervention group; IMNCI, integrated management of childhood illness; IPC, interpersonal counselling; LBW, low birth weight; m, month; MIYCN, maternal infant and young child nutrition; MM, mass media; OR, odds ratio; PA, policy advocacy; PD, prevalence difference; pp, postpartum; PR, prevalence ratio; RR, relative risk; TBA, traditional birth attendant; WAZ, weight-for-age z-score; wk, week; WLZ, weight-for-length z-score

Table S2 Breastfeeding support packages that showed positive effects on breastfeeding outcomes and caregivers' knowledge/skills/practices

**Hospital-based interventions**
**Counselling**
**Ahmadi et al., 2016**<br>**Mothers with premature (34-37wk) infants**<br>**Breastfeeding consultation using BASNEF model and counselling steps using GATHER model**<br>– First session: Consultation and counselling to get mothers familiar with methods of breastfeeding premature infants.<br>– Second session: Counselling to get mothers familiar with several conventional definitions and explanations in breastfeeding.<br>– Third session: Counselling to get mothers familiar with the advantages and disadvantages of natural and artificial feeding of premature infants.<br>– Fourth session: Counselling to get mothers familiar with correct techniques of breastfeeding in premature infants.<br>– Fifth session: Review of the content and conclusion<br>**IG:** 5 sessions of 30 min, 5 consecutive days, **CG:** Conventional training by staff<br>**Delivery:** Researchers, **Follow-up:** 34-37wk gestations to 4m pp
**Aidam et al., 2005**<br>**Pregnant women attending prenatal clinics**<br>**Lactation counselling based on WHO/UNICEF counselling training manual, LINKAGES- "facts for feeding" brochure, Helping Mothers to Breastfeed-book**<br>[Author's developed specific BF materials for the intervention group mothers. These were adapted from-<br>1) the WHO/UNICEF BF counselling training manual<br>2) LINKAGES (Academy for Educational Development) "Facts for Feeding" brochure<br>3) a book entitled Helping Mothers to Breastfeed<br>Intervention group women received education on the following topics at the specified periods: *definition of EBF, early BF initiation, importance of colostrum, on-demand and frequent feeds, benefits of EBF for infant and mother, BF techniques (positioning and attachment, including latch-on), dangers of Prelacteal feeds, adequacy of breast milk for 6m, dangers of breast milk substitutes, and prevention and management of lactation problems (engorgement, sore nipples).*<br>**IG1:** Pre, peri and postnatally, **IG2:** Peri and postnatally, **CG:** Non-BF health education support<br>**Delivery:** Counsellors, **Follow-up:** 3rd trimester-6m pp
**Oliveira et al., 2014**<br>**Adolescent mothers with newborns and their mothers**<br>**Counselling sessions based on WHO guiding principles**<br>[BF counselling session were given in accordance with WHO guiding principles-<br>– BF importance and duration<br>– factors that interfere with the production of milk<br>– the technique of BF<br>– the consequences of using a pacifier and the baby's crying and communication<br>Doubts specific to each mother and grandmother were addressed and clarified.<br>**IG1:** AG not living with mother, with intervention, **CG1:** AG not living with mother, without intervention, **IG2:** AG living with mother, with intervention CG2: AG living with mother, without intervention<br>**Delivery:** Researchers, **Follow-up:** up to 6m

Education

Froozani et al., 1999
Mother-infant pairs
BF education by trained nutritionist (40h BF course)
The BF education was given by a trained nutritionist in a 40-hour breastfeeding course (adapted from Breastfeeding counselling: a training course) in which each mother (after delivery) learned about the advantages of breastfeeding for herself and her child. The topics covered included-

- the anatomy and physiology of the mammary gland
- how to position the infant on the breast and prevent breastfeeding problems
- rooming-in
- breastfeeding on demand
- the importance of exclusive breastfeeding, i.e., using the breast as the only source of nutrients (except for vitamin drops) during the first 4 months of life

IG: Education, face-to-face, after delivery and during follow-up, **CG:** Usual care
Delivery: Trained nutritionist, **Follow-up:** Delivery to 4m

Neyzi et al., 1991
Mother-infant pairs
Educational intervention by paediatric residents
[During the 1st home visit, the mothers were given an appointment card to bring their infants to the University Children's Hospital for follow-up at age 2 weeks. Two paediatric residents were assigned to the care of these infants. The infants were given routine care with subsequent follow-up visits to the hospital at ages 1,2,3 and 4months. The same resident doctor continued to look after the same group of infants throughout study duration. The mothers were also given the doctors' contact details and informed they could call/visit doctors whenever problem arises. During the visits, the doctors spent 5-15 minutes with the mother discussing BF. If needed, the doctors were free to ask the mothers for more frequent visits and to ask them to bring a relative with them.
IG: 2 sessions after delivery, **CG:** Usual care
Delivery: Hospital staff, **Follow-up:** 1,2,3 and 4m

Susin & Giugliani, 2008
Mother-father-infant triads
Educational session by a trained paediatrician
[Intervention included of an educational session about BF by a trained paediatrician. Participants watched an 18-minute video on BF, followed by an open discussion and distribution of an explanatory handout. Both the video and the handout were particularly produced for this study. The video discussed some important aspects of BF, such as-

- the WHO recommendations,
- prevention and management of common BF problems
- the importance of paternal participation

It directly stated that fathers could support the BF mother by helping with household tasks and child care. It also showed several images of fathers helping with household tasks, such as changing diapers, washing dishes, or vacuum cleaning the carpet
IG1: Intervention to mother+father, **IG2:** Intervention to mother only, **CG:** No intervention
Delivery: Paediatrician, **Follow-up:** 1,2,4 and 6m

Ozluses & Celebioglu, 2014
Couples with their infants
Educating fathers

[EBF education was provided to the mothers (20 min/d) in the first intervention group, and to the mothers and fathers (20 min/d for each parent) in the second intervention group; while there was no intervention for the mothers or the fathers in the control group. Education manuals for mothers (28 pages) and fathers (20 pages), prepared by the researchers, were provided to all the parents.
IG1: Mothers-20 min/d, **IG2:** Mothers+fathers-20 min/d, **CG:** No EBF education
Delivery: Researchers, **Follow-up:** 1, 2,4 and 6m

Su & Ouyand, 2016
Pregnant women
Breastfeeding education based on WHO and La Leche League material
The educational materials were selected from WHO and La Leche League International websites. In the intervention group, a ‘‘father support’’ model was used to foster father involvement in decision making with mothers on a feeding model and in supporting BF practices through emotional and physical aspects. The educational intervention was tested by 3 obstetricians, 3 senior midwives, and 3 senior nurses working in an obstetrics unit. The breastfeeding education programme lasted 60-90 minutes, and there were 4-8 participants in each class. PowerPoint presentations, breast models, and newborn dolls were used during the class. Each participant was given a booklet about BF following the education programme.
IG: Education to mother+father, **CG:** Education to mother only
Delivery: Researchers, **Follow up:** 1,2,4 and 6m

Ahmed, 2008
Mothers and preterm infants (born <37wk)
Educational Programme based on PRECEDE model
The 5 session BF educational programme designed using the PRECEDE (Predisposing, Reinforcing, Enabling Constructs in Educational Diagnosis and Evaluation) model included-

- need assessment based on the results of previous studies and surveys, literature review and clinical observation
- specification of programme goals
- formulation of behavioural and learning objectives for each goal
- selection of intervention methods
- specification of time needed to achieve each learning objective
- selecting suitable educational strategies based on Bandura’s SCT
- programme evaluation

IG: 5 session BF education programme, **CG:** Routine care
Delivery: Not clear, **Follow-up:** Birth-3m

Training

Agrasada et al., 2005
Mother-infant (term LBW) pairs
Training programme for counsellors
A 2-tier programme was implemented to select and train the counsellors. An invitation to a postpartum care seminar was sent to key local health centres in 13 cities of Metro Manila. Training material was provided to participants at no cost.

- Level-one training: 3-day seminar on postpartum mother-child health
- Level-two training: Didactic interactive sessions (e.g., role-playing, discussion groups, problem solving) and practical skill workshop (demonstration and return demonstration exercises)

IG1: BF counselling by trained counsellors, **IG2:** Child feeding counselling by trained counsellors, **CG:** Any counselling
Delivery: Counsellors, **Follow-up:** 15d,1,2,3,4,5 and 6m

Promotion
**Cangol & Sahin, 2017**<br>**Pregnant women- applied to pregnancy preparation course**<br>**BF motivation programme based on Pender's Health Promotion Model**<br>The BF motivation programme was structurally based on the conceptual framework of Pender's Health Promotion Model (HPM). The aim of the HPM is to teach individuals behaviours that will improve their health. The main concept of the model is self-efficacy. The components of the HPM are-<br>– behaviour-specific cognitions and effects<br>– perceived benefits and barriers<br>– perceived self-efficacy<br>– activity-related effects<br>– interpersonal influences<br>– situational influences<br>These fundamental components are the main motivating factors in acquiring and maintaining health-promoting behaviours. Four BF motivation programme sessions were held.<br>**IG:** 4 times-antenatal period, 1st postnatal day, 4th-6th postnatal wk and 4th postnatal month,<br>**CG:** Not clear<br>**Delivery:** Researchers, **Follow-up:** 4m
**Gu et al., 2016**<br>**Primiparous women companied by husband/ mother**<br>**Theory of Planned Behaviour (TPB)-based intervention programme**<br>[In addition to routine nursing care, intervention group was offered the theory of planned behaviour (TPB)-based programme. To ensure the quality and consistency of intervention, nurses were trained before the study. The programme comprised 1 individual instruction and 2 group educations at hospital and continued telephone counselling at home during postpartum period.<br>**IG:** Individual instruction, group education and telephone counselling, **CG:** Routine nursing care<br>**Delivery:** Nurses, **Follow-up:** 3d,6wk,4m and 6m pp
**Moudi et al., 2016**<br>**Primiparous women referred to health centre**<br>**BF promotion**<br>To prepare volunteers for support, the researcher held training classes in 5 group sessions with 7-8 persons in each group. Daily sessions over 3 hours with a 45-minute break was held every 90 minutes separately by the investigator at each centre. The education was on the-<br>– definition of peer support- purpose, role, and responsibilities of peer volunteers<br>– communication skills<br>– the basics of breast milk feeding (BMF) (benefits of BMF; anatomy and physiology of lactation; conditions of feeding and breast insertion; proper establishment of BMF; barriers to support and encourage; questions, common concerns, and stories; and false beliefs about BMF)<br>Intervention was delivered in the health care with the lecture approach, using educational slides and images, questions and answers, and role playing.<br>**IG1:** Peer support group (4 times), **IG2:** Health care provider's education (4 training sessions),<br>**CG:** Routine care<br>**Delivery:** N/A, **Follow-up:** 4 and 8wk
**Saljughi et al., 2016**<br>**Pregnant women**<br>**Role playing for promotion of BF**

At first the researcher prepared a scenario for the roles of BF mother, grandmother, aunt and the training midwife according to the instructions by WHO and the latest guidelines by Ministry of Health and Medical Education. The main topics were discussed in two sections:

- the first part was about training the right methods of breastfeeding
- the second part was about the problems during BF (mother's and infant's problems), mother's concerns about infant's nourishment and special needs of each mother during BF

After preparing the scenarios as mentioned, role selection, rehearsal and coordination of role players were conducted at the 35th week of pregnancy (one week before performing).
IG: Training on promoting BF self-efficacy at 36th wk via role playing, **CG:** Routine care
Delivery: Researchers, **Follow-up:** 1m pp

**Community-based interventions**
**Counselling**
**Engebretsen et al., 2014; Tylleskar et al., 2011**<br>**Mother-infant pairs**<br>**EBF counselling based on WHO course**<br>The intervention consisted of EBF counselling by peer counsellors that were from the local communities and trained in a 1wk course by the national research teams with a curriculum adapted from the WHO courses *'Breastfeeding Counselling: A Training Course' and 'HIV & Infant Feeding Counselling: a Training Course'*. All mothers were offered at least 5 home visits, the first occurred in the third trimester of pregnancy.<br>**IG:** Peer counselling (1 antenatal and 4 postnatal), **CG:** Usual care<br>**Delivery:** Trained peer counsellors, **Follow-up:** 3,6,12 and 24wks
**Morrow et al., 1999**<br>**Pregnant women**<br>**Home based peer counselling by trained counselor (La Leche League Mexico)**<br>Three women who had previously worked for the Instituto Nacional de la Nutricion as field data collectors were trained to promote BF as peer counsellors. Each was a resident of San Pedro Martir, aged 25-30 years, had a high-school education, and had a commitment to BF, although they did not necessarily have previous personal BF experience. These peer counsellors were trained and supervised by staff of La Leche League of Mexico and the physician study coordinator, who was also trained in lactation management. The peer-counsellor training consisted of 1 wk of classes, 2 months in lactation clinics and with mother-to-mother support groups, and 1 day of observation and demonstration by visiting experts. Finally, the peer counsellors practised in a neighbourhood nearby San Pedro Martir for 6 months before the intervention trial, during which the content of messages and problem-solving skills were refined.<br>**IG1:** 6 visits, **IG2:** 3 visits, **CG:** No intervention<br>**Delivery:** Researchers, **Follow-up:** 2,4,6wk and 2,3 and 6m pp
**Leite et al., 2005**<br>**Mothers-infants**<br>**Home based peer counselling**<br>Home visits consisted of 6 visits performed by lay counsellors following the discharge from maternity services: on the 5th day from birth, 15th, 30th, 60th, 90th and 120th. These lay counsellors have personal experience in BF their own children and comprise of a workgroup that is associated with the *"Assis Chateaubriand Maternity School"* Milk Bank at the Federal University of Ceara for more than 5 years. Besides the periodic training and supervision activities, all members of this group have participated in a 20-h theory-practice course adapted from *"Breastfeeding counselling: a training course"*.<br>**IG:** Home visits 5,15,30,60,90 and 120d, **CG:** Standard care

**Delivery:** counsellors, **Follow up:** up to 6 m
**Education**
**Aksu et al., 2011**<br>**Pregnant women**<br>**Postnatal BF education based on 18h WHO/UNICEF BF counselling/lactation management course**<br>All women received standard BF education and support from nurses and midwives working at *Zu¨ beyde Hanım Maternity Hospital*. The training topics that were based on the WHO/UNICEF breastfeeding counselling/lactation management courses were introduced to them. Information on the following topic were provided-<br>– production of breast milk<br>– definition of EBF<br>– benefits of EBF for the mother and the child<br>– recommendations for effective breastfeeding<br>– breastfeeding techniques (positioning and attachment)<br>– on-demand and frequent feedings<br>– problems created by pre-lacteals<br>– adequacy of breastfeeding for 6 months<br>– breast milk substitutes<br>– problems connected with BF (e.g. engorgement, tender nipples) and their management<br>**IG:** BF education at home on day 3 postpartum (reinforcement), **CG:** No education/support<br>**Delivery:** Trained supporters, **Follow-up:** 2wk,6wk and 6m pp
**Jakobsen et al., 2000**<br>**Mothers and infants**<br>**Health education based on WHO recommendations**<br>The health education focused on encouraging the mothers to postpone introduction of water and weaning food until the age of 4-6 months according to the WHO recommendation at the time the study was conducted.<br>**IG:** Education provided individual, orally in local language, **CG:** Standard care<br>**Delivery:** Female health worker, **Follow-up:** up to 6m
**Training**
**Bhandari et al., 2003**<br>**Infants**<br>**Promotion of EBF by training existing primary healthcare health and nutrition workers**<br>Health and nutrition workers in the intervention communities attended a 3-day course. Half the course was used for hands-on training in counselling individuals or groups of mothers. The training was based on an adaptation of the Integrated Management of Childhood Illnesses Training Manual on Breastfeeding Counselling, and included training on communication skills, detection of problems with positioning and attachment to the breast and resolving BF difficulties.<br>**IG:** Promotion by CHWs trained in BF (3-day course), **CG:** Usual care<br>**Delivery:** CHWs, **Follow-up:** 3d, 3 and 6m
**Balaluka et al., 2012**<br>**Infants**<br>**Trained CVs -part of community-based nutrition project where CVs were trained in BF**<br>The community volunteers selected were trained at the health district level about the key practices of community nutrition and about promoting BF. Key messages were defined so as to help the mothers understand the importance of EBF from birth-<br>– Breast milk is the first food suitable for newborn babies and young infants

- It protects the infant against diarrhoea and other illnesses
- It strengthens the immunity, without additional costs
- BF strengthens the psycho-emotional relationship between mother and child
- BF repeatedly stimulates breast milk production for the mother

IG: Promoting EBF via door-to-door visits and community meetings, **CG:** Usual care only
Delivery: Community volunteers, **Follow-up:** 3,4,5, and 6m

Mukantwali et al., 2006
Mother-infant pairs
Special training of CHWs based on 40h WHO/UNICEF BF course
A special WHO/UNICEF 40-hour breastfeeding training. The following components were included in this programme-

- counselling and communication
- assessing a breastfed baby,
- expressing breastmilk
- insufficient breastmilk
- refusal to breastfeed
- low birth weight and sick babies
- increasing breastmilk and re-lactation
- building confidence and giving support

IG: Visited by specially trained CHW, **CG:** Visited by normally trained CHW
Delivery: health workers, **Follow-up:** up to 6m

Mukhopadhyay et al., 2017
Mother-infant pairs
Modular training on infant feeding to CHWs in local vernacular
Community health workers- Accredited Social Health Activists (ASHAs) and Female Health Workers (FHWs) of the intervention area were given modular training on IYCF practices in local vernacular. ASHAs were asked to visit the households of study infants fortnightly to counsel the mothers/caregivers about IYCF practices, to help them in solving problems regarding feeding and to give necessary health advices. FHWs counselled the mothers/caregivers at sub-centre and outreach clinics and supported ASHAs during their field visit in promoting optimum infant feeding practices. They were supervised on the job by a group of public health experts. ASHAs and FHWs of the control area did not receive any such training or sensitization. ASHAs and FHWs of both the areas were continuing their routine activities.
IG: Trained community health workers, **CG:** Standard care
Delivery: CHWs, Follow-up: up to 6m

Promotion

Akram et al., 1997
Pregnant women
Promotion of EBF
The intervention consisted of "predefined" health messages that had been pretested on a group of women having the same socioeconomic and literacy status as those in the study group. Messages were pretested with individuals and with groups: the feedback of these women was noted and appropriate changes in the health messages were made. Health education was provided on a one-to-one basis by house visits made by the lady health visitors and health workers. The educators were trained in community motivation and promotion of EBF by resource persons from the lactation management programme. Health messages explained the meaning of EBF, its advantages and stressed the protective role of colostrum and prompt initiation of BF after birth. Mothers were informed about disadvantages of pre-lacteal feeds and the hazards of bottle feeding in promoting

diarrhoea. They were also advised on nutrition and dietary habits and breast care during pregnancy and lactation. These predefined health messages were presented in the form of pictorial flipcharts, photographs representing different BF positions and through verbal communication by trained health workers.
IG: Health messages, **CG:** Not given health message
Delivery: Health workers, **Follow-up:** 1m before delivery-6m pp

Peer support

Bich et al., 2016
Fathers and their pregnant wives from 7-30 wk gestation
Fathers as supporters
To motivate fathers to be involved and act, intervention activities were designed and implemented to target not only fathers but also the social group and general community, including political health care systems. An intervention package was developed consisting of several components:

- mass media communication
- father's group counselling at monthly antenatal and vaccination activities
- home visits by village health workers for individual counselling
- father's role enforcement and community mobilization through public events in collaboration with the Farmers' Association.

The intervention package was integrated within the routine health care services for women and children provided by local health staff within the Chi Linh district health system periphery. Health education campaign and counselling activities targeting fathers were carried out continuously during both antenatal and post-natal periods. The intervention package disseminated key messages on BF through mass media communication using radio, posters and pamphlets, groups counselling sessions and home visits to fathers. A public event entitled *'Who loves their wives and children more'* was also arranged to raise awareness in the community, where teams of fathers competed in front of a live audience answering knowledge questions and performing skits and songs.
IG: BF education material, counselling services at community health centres, invitation to social events and household visits, **CG:** No intervention to fathers
Delivery: CHWs, Follow-up: 4 and 6m
